# Dynamic Alteration of HALP Score as a Predictor in Patients with Receiving Immunotherapy for Advanced Non-Small Cell Lung Cancer

**DOI:** 10.3390/medicina61060989

**Published:** 2025-05-27

**Authors:** Abdülkadir Koçanoğlu, Serdar Karakaya, Esra Zeynelgil, Yakup Düzköprü, Özlem Doğan

**Affiliations:** 1 Department of Medical Oncology, Ankara Atatürk Sanatorium Training and Research Hospital, Ankara 06290, Turkey; drserdarkarakaya@gmail.com; 2 Department of Medical Oncology, Ankara Etlik City Hospital, Ankara 06170, Turkey; esra23.05@hotmail.com; 3 Department of Medical Oncology, Aksaray Training and Research Hospital, Aksaray 68200, Turkey; yakupduzkopru@gmail.com; 4 Department of Medical Oncology, Faculty of Medicine, Altinbas University, Bahcelievler Medicalpark Hospital, Istanbul 34180, Turkey; drozlemdogan@hotmail.com

**Keywords:** HALP score, immunotherapy, lung cancer, pseudoprogression

## Abstract

*Background and Objectives*: This study aimed to investigate the prognostic value of the hemoglobin–albumin–lymphocyte–platelet (HALP) score—a marker reflecting both inflammatory and nutritional status—in patients with metastatic non-small cell lung cancer (NSCLC) undergoing immunotherapy. We also sought to determine whether dynamic changes in the HALP score during treatment could predict therapeutic success and help distinguish between pseudoprogression and hyperprogression. *Materials and Methods*: A retrospective analysis was conducted on 160 patients diagnosed with metastatic NSCLC and treated with immunotherapy at the Ankara Atatürk Sanatorium Training and Research Hospital. Chemotherapy regimens, metastatic sites, baseline and third-month hemograms and biochemistry parameters, and survival data were recorded. Survival outcomes were analyzed using the Kaplan–Meier method with the log-rank test and the Cox proportional hazards regression model using IBM SPSS Statistics. *Results*: The median overall survival (OS) for the entire cohort was 15 months (95% CI: 11.88–18.12). HALP1 score (*p* = 0.048), HALP2 score (*p* = 0.026), and hyperprogression (*p* < 0.001) were statistically significant predictors of OS. Regarding progression-free survival (PFS), the HALP2 score (*p* = 0.031), line of immunotherapy (*p* = 0.046), and hyperprogression (*p* < 0.001) were found to be significant. When comparing patients with increasing versus decreasing HALP scores, those with increasing HALP scores demonstrated significantly better outcomes for both OS (*p* = 0.034) and PFS (*p* = 0.007). *Conclusions*: In patients with metastatic NSCLC undergoing immunotherapy, the HALP score and its dynamic alterations during treatment appear to be non-invasive, easily calculable biomarkers that may predict both OS and PFS.

## 1. Introduction

Lung cancer remains one of the leading causes of cancer-related mortality, particularly in individuals over the age of 50 [1]. Non-small cell lung cancer (NSCLC) accounts for approximately 85% of all lung cancer cases [2]. More than 60% of patients with NSCLC are diagnosed at either locally advanced or metastatic stages [3]. In recent years, targeted therapies, immunotherapy, and chemoimmunotherapy combinations have improved survival outcomes in patients with metastatic NSCLC [4]. However, durable responses to immunotherapy are observed in only 10–40% of patients, and hyperprogression has emerged as a significant clinical challenge in some cases [5]. Hyperprogression and pseudoprogression are circumstances that complicate the evaluation of immunotherapy response. Timely identification of pseudoprogression is particularly important, as it allows for the continuation of immunotherapy in patients who may ultimately benefit. Currently, there are no reliable biomarkers available to predict pseudoprogression. Studies have reported the incidence of pseudoprogression in NSCLC to range between 0.6% and 9.96% [6,7]. This situation highlights the need for biomarkers that can predict immunotherapy response and guide response assessment.

Immunotherapy agents primarily function by enhancing the immune system’s ability to recognize and eliminate cancer cells. Programmed death receptor-1 is a transmembrane protein expressed on cytotoxic T cells, B cells, natural killer cells, monocytes, macrophages, and dendritic cells. When PD-1 binds to its ligand, programmed death ligand-1 (PD-L1), expressed on tumor cells, it suppresses cellular immunity [8]. Currently, PD-L1 expression and tumor mutational burden are the most established biomarkers for predicting response to immunotherapy. However, their predictive accuracy is inconsistent in clinical practice [9,10]. Additional markers—such as tumor neoantigen burden, deficient mismatch repair, microsatellite instability-high, T-cell receptor clonality, tumor-infiltrating lymphocytes, DNA damage and repair genes, and effector T-cell gene signatures—have also been investigated [11,12]. Nonetheless, these biomarkers are costly, tissue-dependent, labor-intensive, time-consuming, and often lack sufficient sensitivity for routine use.

It is well established that systemic inflammatory responses are associated with tumor proliferation, invasion, metastasis, and overall tumor growth [13]. Blood cells interact with tumor cells via cytokine signaling and are, therefore, influenced by the inflammatory milieu [14]. Several indices, including the neutrophil-to-lymphocyte ratio (NLR), platelet-to-lymphocyte ratio (PLR), and systemic immune-inflammation index (SII), have shown prognostic significance in various cancers [15]. In our study, we aimed to evaluate the prognostic role of the hemoglobin–albumin–lymphocyte–platelet (HALP) score, which is a composite marker reflecting both inflammatory and nutritional status, in patients with metastatic NSCLC receiving immunotherapy. We also aimed to investigate whether dynamic alterations in the HALP score during treatment could predict therapeutic efficacy and aid in distinguishing pseudoprogression and hyperprogression.

## 2. Materials and Methods

**Figure 1:**
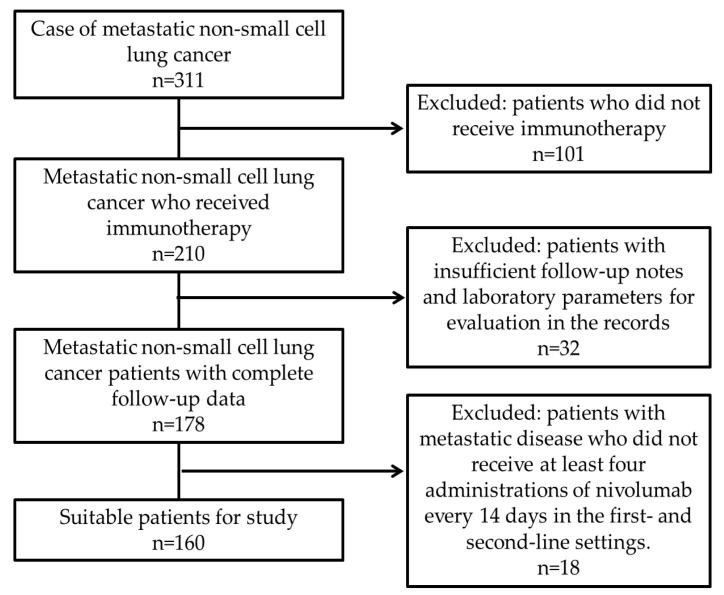
Flow chart showing the patient selection process.

A retrospective analysis was conducted on 311 patients with metastatic NSCLC who were followed up at the medical oncology outpatient clinics of Ankara Atatürk Sanatorium Training and Research Hospital between 1 January 2014, and 1 August 2024. The inclusion criteria for the study were as follows: age over 18 years; a pathologically confirmed diagnosis of NSCLC; receipt of immunotherapy as a first- or second-line treatment at the metastatic stage; receipt of at least four cycles of nivolumab at a standard dose of either 3 mg/kg every 14 days or 240 mg every 14 days; and availability of complete follow-up data in both medical records and electronic systems. Patients were excluded if they had a diagnosis of a second primary malignancy, autoimmune or inflammatory diseases, chronic liver disease, or were undergoing dialysis. Based on these criteria, 160 patients were included in the final (Figure 1).

Collected data included age, sex, smoking and alcohol status, Eastern Cooperative Oncology Group (ECOG) performance status, pathological diagnosis, stage at diagnosis, PD-L1 expression level, comorbidities, prior and subsequent chemotherapy regimens, metastatic sites, baseline and third-month hemogram and biochemical parameters, progression dates, last follow-up dates, and dates of death. Data were retrieved from patient files and electronic medical records.

Overall survival (OS) was calculated from the date of initiation of immunotherapy to either the date of death or the last follow-up. The HALP score was calculated using the following formula: hemoglobin (g/L) × albumin (g/L) × lymphocyte count/platelet count [16]. The score calculated from laboratory values at the start of immunotherapy was referred to as HALP1, while the score based on laboratory data from the third month of treatment was referred to as HALP2. Delta HALP was defined as the difference between HALP2 and HALP1. Based on median values, HALP scores were categorized into two groups: low and high. Patients whose HALP1 score was low but whose HALP2 score was high were classified as increasing HALP, while those whose HALP1 score was high and whose HALP2 score was low were classified as decreasing HALP.

In patients receiving immunotherapy, those who demonstrated disease progression according to RECIST 1.1 at the initial radiological assessment (defined as ≥25% increase in lesion size) but did not develop new lesions consistent with metastasis and were not considered clinically progressive were classified as having pseudoprogression if a response to immunotherapy was observed on follow-up imaging performed 8 weeks later. Conversely, patients whose lesion size increased by ≥50% or who developed new metastatic lesions at the first radiological evaluation during immunotherapy were considered to have hyperprogression [17,18].

Ethical approval for the study was granted by the Ethics Committee of the Ministry of Health Ankara Atatürk Sanatorium Training and Research Hospital (approval number: 2024-BÇEK/214, date: 22 January 2025). The study protocol was prepared in accordance with the Declaration of Helsinki, 1964.

Statistical analyses were performed using IBM SPSS Statistical Software (version 22.0; IBM Corp., Armonk, NY, USA). The clinical and demographic characteristics of the patients were evaluated using descriptive analysis. Categorical and numerical variables were presented as counts and percentages (*n*, %). Data with a normal distribution were expressed as mean ± standard deviation; otherwise, they were reported as median and range. HALP1, HALP2, and delta HALP scores were categorized into two groups, low and high, based on the median values. OS was estimated using the Kaplan–Meier method. Hazard ratios (HR) and 95% confidence intervals (CI) were calculated using the Cox proportional hazards regression model. Differences between groups were evaluated using the log-rank test. Variables that were statistically significant in univariate analysis were included in the multivariate analysis. A *p*-value of <0.05 was considered statistically significant for all analyses.

## 3. Results

A total of 160 patients were included in the study. The median age at diagnosis was 62.5 years (range: 47–79), and the majority of the patients were male (*n* = 134, 83.8%). Regarding pathological subtypes, adenocarcinoma was observed in 77 patients (48.1%), while squamous cell carcinoma was identified in 69 patients (43.1%). At the time of diagnosis, 78 patients (48.8%) presented with de novo metastatic disease. The number of patients diagnosed at stage III, stage II, and stage I were 54 (33.8%), 24 (15%), and 4 (2.5%), respectively. The tumor was most frequently located in the right lung (*n* = 87, 54.4%). The most common site of metastasis was the contralateral lung (*n* = 58, 36.3%), followed by bone metastases (*n* = 25, 15.6%), liver metastases (*n* = 13, 8.1%), and pleural effusion or pleural involvement (*n* = 30, 18.8%). The number of patients with a PDL-1 expression level below 1% was 65 (40.6%). In the initial response assessment, pseudoprogression was observed in 25 patients (15.6%), while hyperprogression was seen in 39 patients (24.4%). Immune-related adverse events were reported in 33 patients (20.6%), with grade 1 toxicities being the most frequently observed (Table 1).

**Figure 2:**
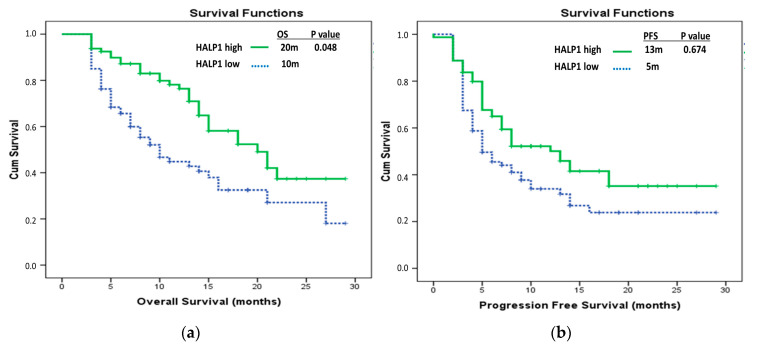
Patients with low and high HALP1 scores: (**a**) comparative overall survival Kaplan–Meier curves; (**b**) comparative progression-free survival Kaplan–Meier curves.

**Figure 3:**
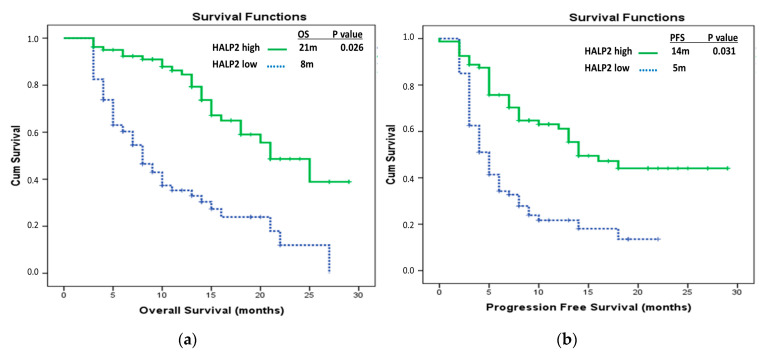
Patients with low and high HALP2 scores: (**a**) comparative overall survival Kaplan–Meier curves; (**b**) comparative progression-free survival Kaplan–Meier curves.

Regarding HALP scores, the median HALP1 score was 22.11, the median HALP2 score was 26.48, and the median Delta HALP score was 2.11. In the entire patient cohort, the median OS was 15 months (95% CI, 11.88–18.12). In the groups with low and high HALP1 scores, the median OS was 10 months (95% CI, 6.62–13.38) and 20 months (95% CI, 14.61–25.40), respectively (*p* = 0.001). For the groups with low and high HALP2 scores, the median OS was 8 months (95% CI, 5.99–10.00) and 21 months (95% CI, 16.31–21.70), respectively (*p* = 0.000). In the groups classified by Delta HALP score, the median OS was 10 months (95% CI, 3.41–16.59) for the low-score group and 18 months (95% CI, 13.52–22.48) for the high-score group (*p* = 0.005). In the multivariate analysis of OS, the HALP1 score (*p* = 0.048), HALP2 score (*p* = 0.026), and hyperprogression (*p* < 0.001) remained statistically significant (Table 2) (Figure 2 and Figure 3).

In the overall patient cohort, the median progression-free survival (PFS) was 8 months (95% CI, 5.66–10.34). In the groups with low and high HALP1 scores, the median PFS was 5 months (95% CI, 2.75–7.25) and 13 months (95% CI, 8.29–17.71), respectively (*p* = 0.027). In the groups with low and high HALP2 scores, the median PFS was 5 months (95% CI, 4.00–6.04) and 14 months (95% CI, 8.53–19.47), respectively (*p* < 0.001). Among patients who received immunotherapy as first-line treatment, the median PFS was not reached, whereas in those who received it as second-line therapy, the median PFS was 5 months (95% CI, 3.65–6.35) (*p* < 0.001). Similarly, in the group without pseudoprogression, the median PFS was 6 months (95% CI, 4.73–7.27), while in the pseudoprogression group, the median was not reached. In the group without hyperprogression, the median PFS was 14 months (95% CI, 10.55–17.46), whereas in the hyperprogression group, the median PFS was 3 months (95% CI, 2.74–3.76) (*p* < 0.001). In the multivariate analysis of PFS, the HALP2 score (*p* = 0.031), line of immunotherapy (*p* = 0.046), and hyperprogression (*p* < 0.001) were found to be statistically significant, while the HALP1 score was not statistically significant (*p* = 0.674) (Table 3) (Figure 2 and Figure 3).

**Figure 4:**
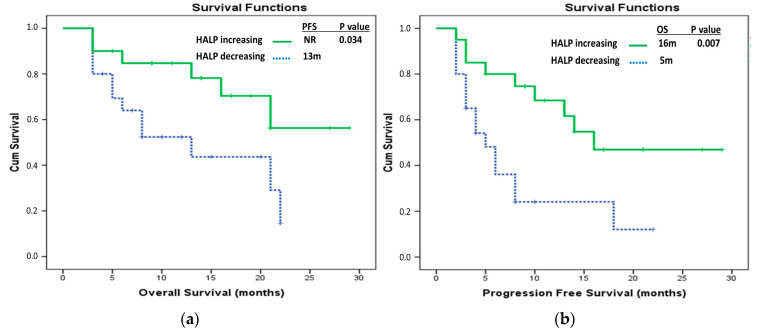
Patients with increasing and decreasing HALP scores: (**a**) Comparative overall survival Kaplan–Meier curves; (**b**) Comparative progression-free survival Kaplan–Meier curves.

There were 20 patients in both the decreasing HALP score and increasing HALP score groups. In the group with a decreasing HALP score, the median OS was 13 months (95% CI, 4.55–21.46), whereas in the increasing group, the median OS was not reached. The median PFS was 5 months (95% CI, 2.65–7.35) in the decreasing HALP group and 16 months in the increasing HALP group. In the analysis conducted within these patient groups, the difference between the groups was statistically significant in favor of the increasing HALP group for both OS (*p* = 0.034) and PFS (*p* = 0.007) (Figure 4).

In the regression analysis investigating the factors predicting pseudoprogression, including age (*p* = 0.329), sex (*p* = 0.345), HALP1 score (*p* = 0.662), and HALP2 score (*p* = 0.346), none of the evaluated parameters were found to be statistically significant. However, in the regression analysis evaluating predictors of hyperprogression that age (*p* = 0.231), sex (*p* = 0.976), HALP1 score (*p* = 0.817), and HALP2 score (*p* = 0.001), only the HALP2 score was statistically significant (OR: 0.94, 95% CI: 0.91–0.98).

## 4. Discussion

In this study, we aimed to investigate the relationship between the pre-immunotherapy HALP score, dynamic changes in the HALP score, and PFS and OS in patients diagnosed with metastatic NSCLC who received immunotherapy. There are retrospective studies in the literature demonstrating the prognostic importance of inflammatory and nutritional markers in patients with metastatic NSCLC [19,20,21,22]. However, to the best of our knowledge, our study is the first to demonstrate an association between the HALP score and both OS and PFS, specifically in metastatic NSCLC patients treated with immunotherapy. It is also the first to show that dynamic changes in HALP scores may be associated with immunotherapy response.

In a study by Cavdar et al., the relationship between OS and inflammatory markers such as NLR, PLR, SII, HALP, and the advanced lung cancer inflammation index (ALI) was examined in 278 patients with metastatic NSCLC. Only 15% of these patients received immunotherapy, while the remainder were treated solely with chemotherapy. In the univariate analysis, NLR, SII, HALP, and ALI reached statistical significance, while in the multivariate analysis, HALP was significantly associated with OS (*p* = 0.002). However, in the subgroup analysis of the small number of patients who received immunotherapy, no inflammatory markers were found to be significantly associated with OS [21]. In another study by Taylor et al., the relationship between preoperative NLR, PLR, SII, HALP, and ALI scores and OS was evaluated in operable NSCLC patients. Only ALI was found to be a significant prognostic factor in the multivariate analysis (*p* = 0.049) [15]. Additionally, a separate study demonstrated that the HALP score had prognostic value for OS in metastatic NSCLC patients treated with chemotherapy [20]. In our study, HALP1, HALP2, and delta HALP were all significantly associated with OS in the univariate analysis. In the multivariate analysis, the HALP1 score, which was measured at the initiation of treatment, was found to be a statistically significant predictor of OS (*p* = 0.048). HALP2, measured in the third month of treatment, showed an even stronger prognostic significance (*p* = 0.026). These findings suggest that dynamic changes in the HALP score may predict OS more effectively than the baseline HALP score in NSCLC patients receiving immunotherapy. The lack of significance for delta HALP in the multivariate analysis may be explained by the calculation method. Patients who initially had high HALP scores that increased further and those who started with low scores that decreased further might both yield similarly low delta HALP values. These two distinct clinical trajectories likely represent different prognoses, yet delta HALP does not differentiate between them. Therefore, we added two subgroups to our analysis: those with initially low HALP scores that increased and those with initially high HALP scores that decreased. When comparing these subgroups, a significant difference in OS was observed in favor of the group with increasing HALP scores (*p* = 0.034).

In the study conducted by Gao et al., it was observed that patients with metastatic NSCLC in the high HALP score group had significantly longer PFS compared to those in the low HALP score group (13 months vs. 9 months, *p* = 0.0038). In this retrospective study, which included 203 patients, all patients received platinum-based doublet chemotherapy [22]. In our study, although the pre-treatment HALP1 score was statistically significant for PFS in the univariate analysis, it did not reach statistical significance in the multivariate analysis. However, the HALP2 score remained statistically significant even in the multivariate model. Patients with higher HALP2 scores were found to have significantly longer PFS (14 months vs. 5 months, *p* = 0.031). Additionally, patients whose HALP scores increased during treatment had longer PFS compared to those whose scores decreased (16 months vs. 5 months, *p* = 0.007). These findings suggest that both the HALP2 score and dynamic change in HALP score during treatment may serve as effective tools to predict PFS.

In our study, a statistically significant difference in PFS was found between patients who received immunotherapy as first-line treatment versus those who received it as second-line (5 months vs. not reached, *p* = 0.046). However, there was no significant difference in OS between these groups.

Pseudoprogression and hyperprogression are clinical situations that complicate response evaluation and treatment management in patients receiving immunotherapy [23]. To guide radiological assessment, iRECIST criteria have been developed [17]. Nevertheless, even with iRECIST, challenges persist in clinical practice. Several studies have investigated methods such as PET/CT patterns or radiomic scoring to predict pseudoprogression and hyperprogression [24,25]. In our study, we evaluated whether the non-invasive HALP score and its dynamic changes could predict pseudoprogression and hyperprogression. Prior studies have reported pseudoprogression rates ranging from 0.6% to 9.96% and hyperprogression rates between 5% and 19.2% in NSCLC patients [6,7,26]. In our study, pseudoprogression was observed in 15.6% and hyperprogression in 24.4% of patients. The higher rates observed may be attributed to our center being a lung-specific hospital, with an experienced team capable of accurately assessing immunotherapy responses both clinically and radiologically. There was no significant difference in OS or PFS between patients with and without pseudoprogression. However, patients who developed hyperprogression had significantly worse OS and PFS. HALP1 and HALP2 scores were not predictive of pseudoprogression. In contrast, the HALP2 score was a significant predictor of hyperprogression. Based on these findings, the HALP2 score may be considered a potential biomarker for predicting hyperprogression in patients undergoing immunotherapy.

The primary limitations of our study are its retrospective, single-center design and the evaluation of patients over a long historical period. As a result, certain clinical characteristics, such as ECOG performance status, comorbidities, and smoking status at treatment initiation, were not adequately obtained from patient records and were therefore excluded from the analyses. To more accurately assess the dynamic changes in HALP scores during immunotherapy and their potential associations, multicenter, prospectively designed studies are warranted.

## 5. Conclusions

In patients with metastatic NSCLC receiving immunotherapy, the HALP score and its dynamic changes emerge as promising, non-invasive, and easily calculable biomarkers for predicting both PFS and OS. The dynamic alteration in HALP score may serve as a valuable guide in evaluating response to immunotherapy.

## Figures and Tables

**Table 1:** Clinicopathological characteristics of patients.

Features	Frequency *n*(%)
Gender	
Female	26 (16.3)
Male	134 (83.7)
Pathology	
Adenocarcinoma	77 (48.1)
Squamous cell carcinoma	69 (43.1)
Adenosquamous carcinoma	2 (1.3)
NOS	12 (7.5)
De novo metastasis	
No	82 (50.2)
Yes	78 (48.8)
Site of metastasis	
Contralateral Lung	58 (36.3)
Bone	25 (15.6)
Liver	13 (8.1)
Brain	9 (5.6)
Pleura/pleural effusion	30 (18.8)
Adrenal gland	19 (11.9)
Distant lymph node	19 (11.9)
Age group	
≤62	80 (50)
>62	80 (50)
PDL-1 groups	
PDL-1 < 1%	65 (40.6)
PDL-1 1–10%	20 (12.5)
PDL-1 10–50%	21 (23.1)
PDL-1 > 50%	20 (12.5)
Missing	34 (21.3)
Pseudoprogression	
No	135 (84.4)
Yes	25 (15.6)
Hyperprogression	
No	121 (75.6)
Yes	39 (24.4)
Line of immunotherapy	
First-line	49 (30.6)
Second-line	111 (69.4)
Immun-related adverse events	
Grade 1	17 (10.6)
Grade 2	13 (8.1)
Grade 3	3 (1.9)

**Table 2:** Analysis of prognostic factors in terms of overall survival.

Features		Univariate Analysis	Multivariate Analysis	
	Median OS (Months, 95% CI)	*p* Value	HR (95% CI)	*p* Value
HALP1 groups				
HALP1 low	10 (6.62–13.38)	0.001 *	Ref 0.58 (0.34–1.00)	0.048 *
HALP1 high	20 (14.61–25.40)			
HALP2 groups				
HALP2 low	8 (5.99–10.00)	0.000 *	Ref 0.472 (0.24–0.91)	0.026 *
HALP2 high	21 (16.31–25.69)			
Delta HALP groups				
Delta HALP low	10 (3.41–16.59)	0.005 *	Ref 0.94 (0.52–1.70)	0.839
Delta HALP high	18 (13.52–22.48)			
Age groups				
≤62	15 (8.80–21.20)	0.782		
>62	16 (13.38–18.62)			
Line of IO				
First-line	27 (14.28–39.72)	0.003 *	Ref 2.86 (1.70–4.82)	0.252
Second-line	14 (11.43–16.58)			
De novo metastasis				
Yes	11 (7.67–14.33)	0.000 *	Ref 0.59 (0.35–1.01)	0.55
No	22 (14.27–29.74)			
Gender				
Female	15 (8.59–21.41)	0.568		
Male	15 (11.64–18.36)			
Pseudoprogression				
No	14 (11.70–16.30)	0.017 *	Ref 0.93 (0.45–1.91)	0.844
Yes	NR			
Hyperprogression				
No	21 (16.56–25.45)	0.000 *	Ref 2.86 (1.70–4.82)	<0.001 *
Yes	5 (3.80–6.20)			

* Represents a significant outcome (*p* < 0.05). CI, confidence interval; HR, hazard ratio; OS, overall survival; NR, not reached; IO, immunotherapy.

**Table 3:** Analysis of prognostic factors in terms of progression-free survival.

Features		Univariate Analysis	Multivariate Analysis	
	Median OS (Months, 95% CI)	*p* Value	HR (95% CI)	*p* Value
HALP1 groups				
HALP1 low	5 (2.75–7.25)	0.027 *	Ref 1.11 (0.69–1.79)	0.674
HALP1 high	13 (8.29–17.71)			
HALP2 groups				
HALP2 low	5 (4.00–6.04)	<0.001 *	Ref 0.506 (0.27–0.94)	0.031 *
HALP2 high	14 (8.53–19.47)			
DeltaHALP groups				
DeltaHALP low	5 (3.56–6.44)	0.002 *	Ref 1.16 (0.67–1.99)	0.6
DeltaHALP high	13 (9.49–16.51)			
Age groups				
≤62	7 (2.67–11.33)	0.791		
>62	8 (5.81–10.19)			
Line of IO				
First-line	NR	<0.001 *	Ref 1.78 (1.01–3.15)	0.046 *
Second-line	5 (3.65–6.35)			
De novo metastasis				
Yes	5 (3.84–6.16)	<0.001 *	Ref 0.82 (0.52–1.30)	0.405
No	14 (6.87–21.14)			
Gender				
Female	6 (3.67–8.34)	0.034 *	Ref 0.66 (0.39–1.13)	0.13
Male	8 (4.03–11.97)			
Pseudoprogression				
No	6 (4.73–7.27)	0.001 *	Ref 0.58 (0.29–1.19)	0.137
Yes	(NR)			
Hyperprogression				
No	14 (10.55–17.46)	<0.001 *	Ref 10.90 (5.91–20.10)	<0.001 *
Yes	3 (2.74–3.76)			

* Represents a significant outcome (*p* < 0.05). CI, confidence interval; HR, hazard ratio; OS, overall survival; NR, not reached; IO, immunotherapy.

## Data Availability

The data generated and/or analyzed in this study are included within this article.

## Abbreviations

The following abbreviations are used in this manuscript:

HALP	Hemoglobin–albumin–lymphocyte–platelet
NSCLC	Non-small cell lung cancer
OS	Overall survival
PFS	Progression-free survival
PD-L1	Programmed death ligand-1
NLR	Neutrophil-to-lymphocyte ratio
PLR	Platelet-to-lymphocyte ratio
SII	Systemic immune-inflammation index
ECOG	Eastern Cooperative Oncology Group
HR	Hazard ratios
CI	Confidence interval
ALI	Advanced lung cancer inflammation index
